# Testing an intergenerational cascade model of adolescent family and individual functioning, young adult Romantic competence, and third-generation child adjustment: Developmental processes and PROSPER intervention effects

**DOI:** 10.1017/S0954579426101473

**Published:** 2026-05-11

**Authors:** Gregory M. Fosco, Mark J. Van Ryzin, Carolyn A. Albright, Seoyoung Chloe Ha, Mark E. Feinberg

**Affiliations:** 1 Human Development and Family Studies, Pennsylvania State University, USA; 2 University of Oregon, USA; 3 Edna Bennett Pierce, Prevention Research Center, Pennsylvania State University, USA

**Keywords:** Child adjustment, family climate, intergenerational mechanisms, parenting, romantic competence

## Abstract

This study evaluated whether young adult romantic relationship quality is an intergenerational mechanism linking Generation 1–2 (G1–G2) family climate and G2 social problem-solving skills during adolescence to G2–G3 parenting and family level-functioning and ultimately G3 child maladjustment and social–emotional competence. Our sample included 396 families with a parent (M_age_ = 28.29; 94% White) from a longitudinal study starting when they were in 6^th^ grade. Participants completed annual assessments through high school, three assessments in young adulthood, and surveys after becoming parents. Two intergenerational pathways emerged: Positive G1–G2 family climates in adolescence predicted less young adult relationship violence; in turn, violence was associated with lower G2–G3 harsh discipline, abusive parenting, and family conflict. Of these, harsh discipline and abusive parenting were associated with G3 children’s adjustment. In addition, G2 social problem-solving skills in adolescence were associated with stronger couple problem-solving skills in young adulthood and with better G2–G3 family routines; in turn, G2–G3 family routines were associated with G3 child social–emotional competence. Finally, moderation effects were observed in which youth who received the PROSPER interventions exhibited associations between adolescent social problem-solving skills and young adult couple problem-solving and G2–G3 parental warmth and (lower) lax discipline.

Developmental and family scientists often seek to understand the life course processes linking the well-being of past and future generations (Haggerty & Carlini, 2020). From a developmental perspective, prospective longitudinal methods can uncover the enduring effects of family and individual processes that reverberate from one generation to the next. From a prevention science perspective, these studies identify possible pathways by which an intervention may reduce risk for future generations and promote societal well-being. In the current study, we evaluated intergenerational linkages linking generation 1–generation 2 (G1–G2) family relationships and generation 2 (G2) early adolescent individual functioning as predictors of later G2 romantic relationship quality in young adulthood, and whether young adult romantic relationship quality sets the stage for providing a more positive (G2–G3) childrearing environment, supporting G3 children’s adjustment.

Prospective, intergenerational studies remain relatively rare in the developmental literature; however, the accumulating evidence points to continuity in parenting behaviors from one generation to the next. Harsh and ineffective parenting in G1–G2 foreshadows risk for harsh, ineffective parenting in the G2–G3 households (e.g., Hops et al., 2003; Simons et al., 1991). Similarly, warm and supportive parenting in the G1–G2 family relationship predicts more warm, supportive G2–G3 parenting (Chen & Kaplan, 2001; Shaffer et al., 2009). These studies offer evidence for what is intuitively true: parenting in one generation sets the stage for parenting of the next. Despite this evidence, it is not always clear *how* parenting is replicated across generations.

Recent studies have begun identifying mechanisms of intergenerational transmission of parenting. For example, problematic parenting and family relations in G1–G2 families are associated with greater risk for externalizing problems in G2 young adulthood, which in turn is associated with poorer parenting and family relationship quality in the next generation (Capaldi et al., 2003; Fosco et al., 2025; Neppl et al., 2009; Rothenberg et al., 2016). Similarly, more positive G1–G2 parenting is associated with better social skills in G2 young adulthood, which in turn is linked with higher levels of G2–G3 parenting competence (Shaffer et al., 2009). Together, these studies suggest that intervening experiences in young adulthood – between the family of origin experiences and childrearing the next generation – may better inform our understanding of intergenerational transmission.

The current study builds on these process models by evaluating an intergenerational cascade model (see Figure 1) in which the G1 family climate and G2 early adolescent problem-solving skills unfold in a transactional process of mutual influence during G2 adolescence. In turn, each of these processes were tested as predictors of G2 young adult romantic relationship quality, in terms of low levels of interpersonal violence, and higher levels of effective relationship conflict resolution. We expected that these young adult romantic relationship experiences would set the foundation for later parenting and family relationship quality, which is ultimately expected to impact the adjustment of the next generation (G3), indicated by greater social–emotional competence and lower levels of psychological maladjustment. Finally, leveraging a sample from the PROSPER community-randomized trial, we evaluated whether the PROSPER-delivered interventions impacted the developmental linkages in these processes.

**Figure 1.**
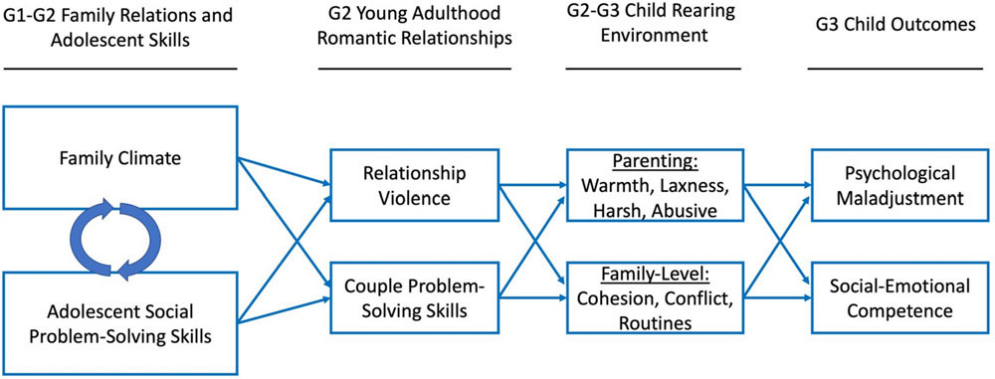
Conceptual intergenerational cascade model.

## A Romantic competence perspective on intergenerational cascades

The successful formation and maintenance of healthy romantic relationships is one of the developmental tasks in young adulthood (Masten, 1995; Sassler, 2010). Young adults (YA) who are in positive, committed relationships tend to have better mental and physical health (Braithwaite et al., 2010; Davila et al., 2017; Golding, 1999; Kamp Dush & Amato, 2005; Williams & Umberson, 2004). Moreover, positive romantic relationships in young adulthood also predict later marital quality (Gottman & Notarius, 2002). In the current study, we focus on two important aspects of romantic relationships in young adulthood that have been identified as particularly robust indicators of long-term outcomes. The first aspect, *relationship violence*, is recognized as a key risk factor for physical and mental health problems, including depression, posttraumatic stress disorder, suicidality, and substance use problems (Campbell, 2002; Campbell & Lewandowski, 1997; Coker et al., 2002; Golding, 1999; Stubbs & Szoeke, 2021). The second aspect is *couple problem-solving skills*, which refers to using positive strategies for resolving disagreements, including remaining calm, listening, and working toward a mutually beneficial solution to problems. Couple problem solving skills are associated with relationship health, longevity, and satisfaction (Conger & Rueter, 1999; Gottman & Notarius, 2002; Zhou & Davila, 2019). These are two important aspects of a “romantic competence” hypothesis – that couples who are low in interpersonal violence and high in couple problem-solving skills (Davila et al., 2007) – would be positioned to be more effective in their parenting and experience more positive family relations in the G2–G3 childrearing environment. Placing romantic competence within the developmental cascade, we must consider the developmental precursors and sequelae of romantic competence.

## Developmental foundations of Romantic competence

The Development in Early Adult Romantic Relationships theoretical model (DEARR; (Bryant & Conger, 2002) offers helpful guidance in conceptualizing the developmental processes underlying young adult romantic competence. Importantly, the DEARR model highlights the importance of youth individual characteristics and family of origin relations as developmental precursors to romantic competence in young adulthood. In the current study, we focus on adolescent social problem-solving skills and the quality of the family climate in adolescence as key factors that may predict young adult romantic competence.

The DEARR model underscores *social problem-solving skills* as a characteristic of YA that promotes young adult romantic competence (Bryant & Conger, 2002). Following this postulation, we extend the DEARR model by evaluating social problem-solving skills in adolescence as a precursor to young adult romantic competence, drawing on the larger social–emotional competence literature, which points to early life social–emotional competence as having long-term implications into adulthood (e.g., Jones et al., 2015). Specifically, we focused on adolescent problem-solving skills. Often applied in social contexts (e.g., peer conflicts), adolescent social problem-solving skills encompass a tendency to gather information when facing a challenging situation or problem to gather information, identify possible responses to the situation, consider the consequences of the different responses, and choose the appropriate solution (Scheier & Botvin, 1998). Adolescents who are adept in using effective problem-solving skills tend to exhibit lower levels of aggression, delinquency, and other problem behaviors (Jaffee & D’Zurilla, 2003). In addition, adolescents with better problem-solving skills are at lower risk for alcohol and marijuana use (Jaffee & D’Zurilla, 2009). Related to the current study, early adolescents’ problem-solving skills also predict better family relationships in middle adolescence (LoBraico et al., 2022; Siu & Shek, 2010). Social problem-solving skills are often learned and applied in interpersonal contexts and may promote more effective problem-solving skills in later romantic relationships, resulting in more constructive conflict behaviors and reduced risk for violence (Bryant & Conger, 2002). In the current study, we expected that social problem-solving would be associated with better romantic competence, evidenced by reduced risk for relationship violence and with better couple conflict problem-solving.

A second developmental precursor to young adult romantic competence is G1–G2 family relationship quality. As noted in the DEARR model, G1–G2 family relationship quality also may play a direct role in promoting or inhibiting the development of young adult romantic competence (Bryant & Conger, 2002). A direct link between G1–G2 family relationships and romantic competence is consistent with the view that family relationships establish patterned interactions that, over frequent repetition and rehearsal, can become habitual ways of engaging with family members and replicated in relationships outside of the family (Minuchin, 1974). Empirical examples are found in coercive family process research: family interaction patterns characterized by escalating hostility and aggression in the family predict aggressive behavior in other relationships outside of the family (Patterson, 2015). Consistent with this view, warm, nurturant parenting practices, or harsh parenting in adolescence both are linked with young adult romantic competence (e.g., Labella et al., 2018; Spilman et al., 2013; Xia et al., 2018).

Other work points to family-level relationship quality in adolescence as a particularly salient predictor of young adult mental health (e.g., Fosco et al., 2012) and young adult romantic relationship quality. Importantly, a positive family climate in adolescence is a robust predictor of young adult romantic competence, even when accounting for adolescents’ individual functioning, such as hostile-aggressive behavior, assertiveness, and positive engagement within the family (Fosco et al., 2016; Xia et al., 2018), or deviant peer relationships (Ha et al., 2019). Thus, we hypothesized that family climate in adolescence would be directly associated with young adult relationship violence and couple problem-solving skills. However, as noted in the DEARR model, family climate and adolescent social problem-solving may be linked (Bryant & Conger, 2002). Thus, we also modeled possible bidirectional relations among these processes in adolescence (see Figure 1).

## Young adult Romantic competence as a pathway of intergenerational transmission

We consider G2 young adult romantic competence as a developmental pathway linking G1–G2 family and individual functioning and G2-G3 parenting and family-level relationship quality. Central to this notion is an interpersonal relationship transmission process: that G1–G2 family relationships and adolescent social-problem solving skills set the stage for more successful interpersonal qualities in young adult romantic relationships, through which interpersonal skills are further rehearsed and refined. In turn, young adult romantic relationships lay the foundation for later G2–G3 family interactional patterns in the childrearing environment. Thus, we expect that young adult romantic competence would predict the quality of parenting and family relationships in the G2–G3 environment, ultimately supporting or undermining G3 children’s adjustment.

There is some preliminary support for the romantic competence pathway of intergenerational transmission. One study documented links between G1–G2 family conflict predicting late-adolescent externalizing problems; in turn, G2 young adult externalizing problems are associated with elevated G2–G3 family conflict (Rothenberg et al., 2016). These findings support the view that high-conflict homes may foster late adolescents’ aggressive, problematic interpersonal behaviors that then are generalized in the G2–G3 family interactions.

A more direct test of the romantic competence hypothesis is found in three intergenerational samples documenting links between G1 parenting and family experiences with G2 romantic competence, and G2–G3 childrearing. The first study, from the Family Transitions Project is a prospective intergenerational sample of approximately 290 adolescents from families in Iowa, and used high-quality assessments including survey, interview, and observational methods (Conger et al., 2013; Schofield et al., 2017). Findings from this study suggest that harsh G1–G2 parenting during G2 adolescence was associated with poorer G2 young adult romantic relationship quality; in turn, G2 relationship quality was negatively associated with G2–G3 harsh parenting. A second study demonstrated couple interpersonal violence as a mediator of parents’ history of maltreatment and next-generation adolescent maltreatment risk (Adams et al., 2019). The third study, leveraging the Minnesota Longitudinal Study sample (LaBella et al., 2019) reports that G1–G2 childhood experiences of abuse and neglect predicted less positive romantic relationship quality in young adulthood; in turn, less positive G2 romantic relationship quality was associated with lower levels of G2–G3 supportive parenting. In addition, G1–G2 childhood experiences of abuse and neglect also were associated with G2 young adult relationship violence, and in turn, relationship violence was associated with increased risk for G2–G3 Child Protective Services involvement (LaBella et al., 2019). This study revealed that there may be different effects of positive couple functioning and couple violence for later outcomes, underscoring the value of examining positive relationship qualities (e.g., effective couple problem-solving skills) and risk processes (i.e., relationship violence) as distinct pathways of risk or promotion.

## The current study

Compelling evidence suggests that young adult romantic relationships may be a pathway of intergenerational transmission of G1–G2 family environment and G2–G3 outcomes; however, several important gaps remain that we address in the current study. First, this literature has focused on parenting quality across generations. Yet the family environment is multifaceted, and different facets may have differential impacts on offspring. For example, family climate is distinct from parenting practices (Dishion et al., 2000; Fosco & Grych, 2013; Morris et al., 2007) and has unique implications for young adult romantic competence, even when accounting for the effects of effective parental discipline practices (Xia et al., 2018). In the current study, we examined the role of the G1–G2 family climate for G2 young adult romantic competence. Second, the effects of adolescent individual functioning on family relationships is largely overlooked in family research, despite evidence of child effects on family functioning (e.g., Crouter & Booth, 2003). In our developmental cascade model (see Figure 1), we evaluated the reciprocal effects of individual and family functioning during adolescence, while also evaluating the distinct predictive effects of G1–G2 family climate and G2 adolescent social problem-solving on G2 young adult romantic competence. Third, most studies focus narrowly on one or two G2–G3 family outcomes (e.g., harsh parenting), obscuring the degree to which young adult romantic competence may have wide-ranging impacts on multiple domains of parenting or family relationship quality. In our study, we conduct analyses to predict G2–G3 warm, lax, harsh, and abusive parenting simultaneously; and in our second model, we predict G2–G3 family cohesion, family conflict, and family routines. By including a wide range of G2–G3 family qualities, it is possible to gain a more nuanced assessment of the developmental linkages over time. Fourth, intergenerational studies examining romantic competence pathways have not evaluated longitudinal links between G2–G3 parenting and family functioning with G3 child adjustment. In the current study, we conduct prospective analyses predicting G3 child maladjustment and social–emotional competence from G2–G3 parenting and family climate.

As a final aim, the current study evaluated whether the proposed developmental cascades may be tempered by evidence-based universal prevention programs delivered in early adolescence. Recent findings suggest that other programs, namely the Fast Track intervention, have been found to moderate the developmental linkages across three generations (Gorla et al., 2025). Their study documents a disrupted developmental cascade of parental depression, in which G1 depression predicted G2 depression, and G2 depression was associated with G3 internalizing problems, but only for youth in the control group. Youth who received the Fast Track interventions did not exhibit continuity in the G2–G3 depression risk link. Thus, randomized trials of intervention programs may exhibit protective effects on subsequent generations by disrupting the developmental links among risk factors.

The current study followed a sample of adolescents in 6^th^ Grade, who were randomized to receive the PROSPER interventions or school programming as usual. Most of those assigned to the PROSPER condition participated in an evidence-based school program and their families were invited to participated in a family-centered program (see Spoth et al., 2004 for more details). In the current study, we evaluated whether the developmental cascades tested in this study were disrupted or enhanced by the PROSPER-delivered interventions, consistent with prior work (Gorla et al., 2025).

## Method

This study was conducted using data from the Pathways to Health (P2H; R01 HD092439, PIs Fosco & Feinberg) project, which was conducted as an extension of the PROSPER (Promoting School-Community–University Partnerships to Enhance Resilience; R01 DA013709, PI: R. Spoth; Spoth et al., 2004) community-randomized trial. The PROSPER trial was conducted to evaluate the effectiveness of the evidence-based preventive intervention delivery system, leveraging a community partnership model. The randomized trial recruited 28 rural or semi-rural community school districts in Pennsylvania and Iowa that either received family-and school-based intervention programs in Grades 6 and 7, or a control condition (Spoth et al., 2007; Spoth et al., 2007 for details on programs and delivery). Two cohorts of 6^th^ grade youth participated in school-based surveys beginning in the 2002–2003 and 2003–2004 school years. The current study used data collected from a sample of 10,845 students in the Fall term of Grade 6 (Wave 1; M_age_ = 11.8) and Spring terms of Grades 6 (Wave 2; M_age_ = 12.4), 7 (Wave 3; M_age_ = 13.2), 8 (Wave 4; M_age_ = 14.3), 9 (Wave 5; M_age_ = 15.2), and 10 (Wave 6; M_age_ = 16.2). YA were randomly sampled after high school from the original trial (*N* = 1,984; 54% female), with oversampling for baseline risk factors, such as conduct problems. In the YA sample, 1,008 participants reported currently being in a committed relationship, and thus were the subsample of participants who responded to surveys related to romantic competence. YA data collection occurred over three measurement occasions, when participants were average ages 19.5, 22.8, and 24.6. Eighty percent of the YA sample was retained at the third occasion (88% completed at least 2 of 3 YA assessments). The three YA occasions were averaged together to represent this developmental period.

The P2H study began in 2018 recruiting participants who had become parents (*N* = 396). The original design called for children who were at least 1.5 years old; most were first-born children (64.3%). G3 children (52% boys) of the P2H G2 participants were an average of 3.98 years old (SD = 2.47) at the first G2-G3 assessment (“Wave A”) and an average of 5.32 years old (SD = 2.66) at the second G2–G3 assessment (“Wave B”; when child adjustment was analyzed). Parents (M_age_ = 28.29, SD_age_ = 1.39 at Wave A) identified as the child’s mother (70.2%), father (26.8%), stepparent (2.3%) or other caregiver (0.8%); most of the sample were biological parents to the target child (95.4%). Their racial background was White (94%), Black (3%), or other races (3%). Most G2 parents (85%) reported living with another caregiving adult in the home. G2 parent education levels included high school graduates (23%), some college (30.3%), college graduates (30.1%), or earning a graduate degree (16.7%). G2 reported household incomes distributed: less than $40,000 per year (26.6%), between $40,000 and $60,000 per year (17.6%), between $60,000 and $80,000 a year (16.2%), between $80,000 and $100,000 per year (15.9%), and over $100,000 per year (20.7%). G2 parent’s age at birth of their child was 24.3 years old, on average (SD = 2.92).

### Sample comparisons and attrition analysis

We compared our intergenerational sample (*n* = 396) with: (a) the remainder of the full PROSPER sample (*n* = 10,449) and (b) the remainder of the YA subsample (those in romantic relationships) (*n* = 612) on all variables used in the analytic model. Small sample differences emerged with the full PROSPER sample on two of twelve variables collected: adolescent social problem-solving skills at Wave 2 (*d* = 0.11), suggesting slightly higher skills for those who did participate in the intergenerational study, and relationship violence in young adulthood (*d* = −0.23), suggesting the intergenerational sample exhibited slightly higher rates of YA relationship violence. We also compared differences among those in the YA sample who were in a romantic relationship in young adulthood to compare those who did and did not participate in the intergenerational study. Of the twelve comparisons, two were significant, for adolescent social problem-solving skills at Waves 3 (*d* = 0.17) and 4 (*d* = 0.23). No other differences were observed.

### Measures

#### G1-G2 adolescence

##### Adolescent family climate

Adolescents reported on the quality of the family climate by responding to 7 items from the Family Environment Scale (Moos & Moos, 1994). These items tapped into family cohesion (e.g., “family members really help and support each other”), conflict (e.g., “we fight a lot in our family”), and organization (e.g., “we are generally very neat and orderly”). Items were rated on a 5-point scale, from *strongly agree* (1), *agree* (2), *neutral or mixed* (3), *disagree* (4), or *strongly disagree* (5). The scale was scored so that higher values indicated more cohesion, organization, and less conflict in the family. Internal consistency was acceptable (α = 0.74–0.82 across Waves 1–6).

##### Adolescent social problem-solving skills

Adolescent problem-solving skills were measured with 5 items from the Coping Assessment Battery (Bugen & Hawkins, 1981; Trudeau et al., 2003) that asked adolescents how they typically handle problem situations, including their ability to gather information, weigh options and consider risks and consequences. The response scale ranged from *Never* (1) to *Always* (5). An example item is “When you have a problem, how often do you think about the consequences of each choice” (α = 0.90–0.93 across Waves 1–6).

#### G2 young adult Romantic competence

##### YA couple problem-solving

YA completed the 7-item cooperative problem-solving measure (Assad et al., 2007) and one additional item (8 items total): “How often do you have good ideas about how to solve the problem?” YA reported on their own behaviors when they have a problem to solve with their partner. The response scale ranged from *Always* (1) to *Never* (7). An example item is “…listen to your partner’s ideas about how to solve the problem.” Scale reliability was acceptable (α =0.79).

##### YA relationship violence

Relationship violence was measured with 11 items drawn from the physical violence subscale from the Conflict Tactics Scale (Straus, 1979). YA rated the frequency at which they perpetrated different types of interpersonal violence toward their partner in the past year. The response scale ranged from 0 (zero times) to 6 (more than 20 times). An example item is “…throwing things at your partner.” Scale reliability was acceptable (α = 0.86).

#### G2-G3 parenting

##### Warmth

G2 parental warmth toward G3 child was measured using 8 items from the Parental Attitudes toward Child Rearing (Easterbrooks & Goldberg, 1984). Each item was rated on a 6-point scale from *strongly disagree* (1) to *strongly agree* (6), with higher scores indicating greater warmth (e.g., “I respect the child’s opinions and encourage him or her to express them”). Scale reliability was acceptable (α = 0.68).

##### Lax discipline

G2 parent’s lax discipline was measured using 9 items from the Parenting Scale (Arnold et al., 1993). Each item was rated on a 7-point scale, with higher scores indicating more lax parenting behaviors (e.g., “I threaten to do things that I know I won’t actually do”). Scale reliability was good (α = 0.85).

##### Harsh discipline

G2 parents’ harsh discipline was measured using 7 items from the Parenting Scale (Arnold et al., 1993). Items were rated on a 7-point scale with higher scores indicating more harsh discipline (e.g., “When my child does something I don’t like, I insult my child, say mean things, or call my child names”). Scale reliability was acceptable (α = 0.68).

##### Abusive parenting

G2 parental psychological and physical abuse over the past year was measured using 12 items from the Parent–Child Conflict Tactics Scales (Straus et al., 1998), rated on a 7-point scale from *never* (0) to *more than twenty times* (6). Sample items include, “I slapped my child on the hand, arm, or leg,” “I shouted, yelled, or screamed at my child.” Higher scores indicated more frequent abusive behaviors. Scale reliability was acceptable (α = 0.78).

#### G2–G3 family-level functioning

##### Family conflict

Family conflict was assessed using 6 items from the shortened Family Environment Scale (Bloom, 1985). Items were rated on a 5-point scale, ranging from *almost never* (1) to *always* (5). Sample items include, “Family members expressed anger at one another,” “Family members quarreled.” Higher scores reflect higher levels of conflict. Scale reliability was acceptable (α = 0.88).

##### Family cohesion

Family cohesion was assessed using 5 items from the shortened Family Environment Scale (Bloom, 1985), rated on a 5-point scale, from *almost never* (1) to *always* (5). Sample items include, “Family members really helped and supported one another,” “Family members got along well with each other.” Higher scores reflect higher levels of cohesion. Scale reliability was acceptable (α = 0.85).

##### Family routines

Family routines were measured using 11 items from the Child Routines Inventory (Sytsma et al., 2001), rated on a 5-point scale from *never* (0) to *nearly always* (4). Sample items include, “My child eats at least one meal a day with the family,” “Family has a certain “family time” each week when they do things together at home.” Higher scores reflect greater regularity of family routines. Scale reliability was acceptable (α = 0.82).

#### G3 child maladjustment

##### Externalizing and internalizing problems

Children’s externalizing and internalizing problems were measured using externalizing and internalizing problem items from Child Behavior Checklist (Achenbach & Rescorla, 2000, 2001). Given the range in age, different versions were used (1.5–5 and 6–18), and these externalizing (1.5–5: α = 0.90, 6–18: α = 0.90) and internalizing (1.5–5: α = 0.88, 6–18: α = 0.89) scales demonstrated acceptable reliability. Scores were converted into externalizing and internalizing T-scores to allow for comparable scores across all children for analysis.

##### Child behavior problems

Children’s behavior problems were measured 36 items from the Eyeberg Child Behavior Inventory (Burns & Patterson, 1990), on a 5-point Likert scale from *Never* (1) to *Always* (5). Sample items include “Does not obey house rules on own,” “Physically fights with friends own age,” and “Physically fights with friends own age.” Scale reliability was acceptable (α = 0.92).

#### G3 child social–emotional competence

***Social Emotional Competence*** was measured with three scales. The first two scales (social and emotional competence and prosocial behaviors) were drawn from the social competence scale developed by the Conduct Problems Prevention Research Group (CPPRG, 1995), with items rated on a 4-point Likert scale from *Not at all* (1) to *Very Well* (4). The *social and emotional competence* scale (labeled “emotion regulation” by the scale developers) included 7 items: “Accepts things not going his/her way,” “Thinks before acting,” “Give suggestions without being bossy,” and “Listens to others’ point of view,” “Understands others feelings, “Identify feelings appropriately,” and “Recognize the needs of others”. Scale reliability was good (α = 0.87). For clarity, we refer to this scale as social and emotional competence, to more closely align what is captured in the scale and avoid confusion with other established measures of emotion regulation. The *prosocial communication* scale included 7 items, including “Act helpful to others” and “listen to others’ points of view,” and “act friendly toward others (α = .88). The third scale, *empathic behaviors*, was measured using the MacArthur Health and Behavior Questionnaire (Armstrong & Goldstein, 2003). Each item was rated on a 5-point scale, from (1) *Never* to (5) *Always*. Sample items include “In the past month, please describe how often your child offer help to other children who are having difficulty.” Scale reliability was acceptable (α = 0.83).

### Covariates

**G2 parent sex** was coded as 1 (female), 2 (male), or 3(something else better describes me). Only one participant endorsed “something else better describes me” making it impossible to analyze as a separate value. Thus, this variable was included as a dichotomous covariate. G3 **child age** was scored as years. G2-G3 annual **family income** was reported in $10,000 increments, from 1 ($0–9,999) to 11 ($100,000 or more).

## Analysis plan

After calculating descriptive statistics and correlations, we computed structural equation models using methods extending the Random Intercept Cross-Lagged Panel Model (RI-CLPM; Mulder & Hamaker, 2021) to simultaneously evaluate: (a) reciprocal within-person relations between G1 family climate and G2 adolescent social problem-solving skills, (b) whether the random intercepts for G1–G2 family climate and adolescent social problem-solving skills predicted G2 YA romantic competence (relationship violence and couple problem-solving), (c) and whether G2 romantic competence predicted G2–G3 childrearing environment and ultimately G3 adjustment (maladjustment and social–emotional competence). In addition, paths were estimated linking random intercepts in G1–G2 to G2–G3 childrearing environment; random intercepts and YA romantic relationship variables also predicted G3 child adjustment.

Two models were computed, to evaluate different aspects of the G2-G3 childrearing environment. In the first model – the parenting model – we evaluated four parenting dimensions (i.e., abusive parenting, harsh discipline, lax discipline, and warmth) as unique pathways linking YA romantic competence to G3 child adjustment. In the second model – the family-level model – we evaluated family-level conflict, cohesion, and routines as unique pathways linking YA romantic competence with G3 child adjustment. In both models, we included covariates from the G2–G3 assessment: G2 parent sex, G3 child age, and G2–G3 family income as covariates. G2–G3 childrearing environment factors and G3 adjustment outcomes all were regressed on covariates; covariates were also allowed to correlate with YA romantic competence and random intercepts. Model fit was determined to be acceptable when meeting conventional fit indices: Root Mean Square Error of Approximation (RMSEA) < 0.08, the Bentler Comparative Fit Index (CFI) > 0.90, the Tucker–Lewis Index (TLI) > 0.90, and the Standardized Root Mean Square Residual (SRMR) < 0.08 (Hu & Bentler, 1999). Finally, we conducted post-hoc tests to evaluate whether the overall model was a good representation of the sample, by conducting tests of moderation by groups. First, we evaluated whether predictors of G2-G3 family climate or parenting differed as a function of the G3 child being the first-born or not. Second, we evaluated whether PROSPER interventions impacted the nature of relations among variables in the model by comparing intervention participants with those in the control groups. Because of model complexity, multiple group comparisons could not be conducted, so we added interaction terms for all key model paths; these interaction terms were evaluated for statistical significance.

## Results

We provide descriptive statistics and correlations in Table 1. Three potential covariates from Wave A were found to be significantly correlated with outcomes (i.e., parent sex, child age, and family income) and were included in the model; all other covariates were excluded to preserve statistical power.

**Table 1.** Correlations, means, and standard deviations

	1	2	3	4	5	6	7	8	9	10	11	12	13	14	15	16	17	18	19	20	21	22	23	24	25	26	27
1. Family Climate (W1)	–																										
2. Family climate (W2)	**0.59**	–																									
3. Family climate (W3)	**0.49**	**0.58**	–																								
4. Family climate (W4)	**0.44**	**0.52**	**0.63**	–																							
5. Family Climate (W5)	**0.38**	**0.45**	**0.56**	**0.65**	–																						
6. Family climate (W6)	**0.35**	**0.41**	**0.50**	**0.59**	**0.67**	–																					
7. Prob solving (W1)	**0.47**	**0.39**	**0.31**	**0.27**	**0.22**	**0.19**	–																				
8. Prob solving (W2)	**0.39**	**0.50**	**0.37**	**0.31**	**0.26**	**0.23**	**0.5 1**	–																			
9. Prob solving (W3)	**0.31**	**0.38**	**0.51**	**0.38**	**0.32**	**0.28**	**0.3 9**	**0. 49**	–																		
10. Prob solving (W4)	**0.27**	**0.32**	**0.38**	**0.49**	**0.39**	**0.32**	**0.3 3**	**0.4 0**	**0.5 1**	–																	
11. Prob solving (W5)	**0.23**	**0.26**	**0.30**	**0.35**	**0.44**	**0.33**	**0. 29**	**0. 35**	**0. 4 3**	**0. 5 6**	–																
12. Prob solving (W6)	**0.18**	**0.21**	**0.25**	**0.28**	**0.32**	**0.39**	**0. 24**	**0.3 1**	**0. 37**	**0.4 8**	**0.5 4**	–															
13. Prob solving (YA)	**0.17**	**0.17**	**0.21**	**0.21**	**0.19**	**0.20**	**0.1 4**	**0. 18**	**0.1 8**	**0. 20**	**0. 22**	**0.18**	–														
14. Rel. vio. (YA)	**−0.14**	**−0.13**	**−0.16**	**−0.19**	**−0.20**	**−0.19**	**−.0 12**	**−0.1 3**	**−0.1 5**	**−0.1 3**	**−0.15**	**−0.1 3**	**−0.46**	–													
15. Warm par (A)	0.10	0.11	0.10	0.09	**0.11**	0.00	**0.18**	**0.23**	**0.23**	**0.26**	**0.23**	**0.21**	**0.27**	−0.03	–												
16. Lax Discipline (A)	**−0.18**	**−0.21**	−0.09	**−0.18**	**−0.11**	−0.02	**−0.13**	−0.09	**−0.11**	**−0.14**	**−0.11**	**−0.14**	**−0.24**	**0.16**	**−0.21**	–											
17. Harsh discipline (A)	**−0.12**	**−0.25**	−0.11	**−0.15**	**−0.12**	−0.08	−0.10	**−0.19**	**−0.16**	**−0.18**	**−0.15**	**−0.15**	**−0.30**	**0.25**	**−0.33**	**0.33**	–										
18. Abusive par (A)	**−0.12**	**−0.17**	−0.09	−0.07	**−0.14**	−0.04	−0.08	−0.06	−0.09	**−0.14**	**−0.15**	**−0.16**	**−0.21**	**0.32**	**−0.11**	**0.12**	**0.45**	–									
19. Family conflict (A)	**−0.19**	**−0.20**	**−0.21**	**−0.24**	**−0.22**	**−0.17**	**−0.14**	−0.09	**−0.12**	**−0.13**	**−0.15**	0.00	**−0.20**	**0.28**	−0.09	**0.16**	**0.26**	**0.24**	–								
20. Family coh (A)	**0.12**	**0.16**	**0.12**	**0.13**	**0.18**	0.07	**0.16**	**.013**	**0.12**	**0.12**	0.09	−0.02	**0.18**	**−0.12**	**0.30**	**−0.30**	**−0.27**	**−0.12**	−0.**37**	–							
21. Family rout (A)	**0.23**	**0.25**	**0. 21**	**0.22**	**0.25**	**0.21**	**0.24**	**0.20**	**0.20**	**0.24**	**0.20**	**0.19**	**0.22**	**−0.15**	**0.39**	**−0.32**	**−0.29**	**−0.22**	**−0.28**	**0.37**	–						
22. Internalizing (B)	**−0.11**	−0.10	**−0.12**	**−0.20**	**−0.14**	**−0.1 5**	0.00	−.008	−0.10	−0.06	−0.09	−0.10	**−0.1 9**	**0. 16**	−0.04	**0.12**	**0.2 3**	**0.1 3**	**0.15**	**−0.2 0**	**−0.1 3**						
23. Externalizing (B)	**−0.15**	**−0.17**	**−0.16**	**−0.21**	**−0.14**	**−0.2 0**	−0.01	−0.09	−0.09	−0.09	**−0.1 5**	−.11	**−0.17**	**0.1 2**	−0.04	0.09	**0.21**	**0.2 6**	**0.1 8**	**−0.1 6**	**−0.2 2**	**0. 69**					
24. Beh problems (B)	**−0.12**	**−0.13**	−0.08	**−0.16**	**−0.12**	**−0.13**	0.00	−0.08	−0.02	−0.08	−0.09	−.03	**−0.19**	**0.18**	0.04	0.09	**0.23**	**0.31**	**0.24**	**−0.21**	**−0.14**	**0.6 0**	**0.77**	–			
25. Soc Emo com (B)	**0.15**	**0.18**	**0.13**	**0.18**	0.11	**0.14**	0.10	**0.12**	0.07	0.10	0.11	.03	**0.20**	**−0.12**	**0.17**	**−0.19**	**−0. 27**	**−0.21**	**−0.22**	**0.29**	**0.31**	**−0.4 0**	**−0.5 7**	**−0.62**	–		
26. Prosocial com (B)	**0.18**	**0.18**	**0.11**	**0.18**	0.10	0.10	0.11	**0.12**	0.04	**0.12**	0.07	.03	**0.17**	−0.10	**0.11**	**−0.18**	**−0.19**	**−0.15**	**−0.18**	**0.23**	**0.27**	**−0.3 9**	**−0.5 0**	**−0.54**	**0.86**	–	
27. Empathetic beh (B)	0.09	**0.13**	0.08	**0.14**	0.06	0.05	0.08	**0.1 3**	0.09	0.09	0.09	**0. 18**	**.1 3**	−0.01	**0.22**	**−0.17**	**−0.18**	−0.07	−0.07	**0.20**	**0.27**	**−0.1 8**	**−0.2 2**	**−0.17**	**0.41**	**0.44**	–
*N*	390	347	350	352	351	310	371	349	352	354	350	309	360	361	385	380	381	381	384	386	387	338	341	347	352	352	348
*M*	3.62	3.63	3.49	3.37	3.32	3.29	3.77	3.56	3.44	3.41	3.49	3.54	5.74	.071	5.31	2.73	2.41	0.76	2.23	4.29	3.31	47.67	47.19	2.03	2.82	3.01	3.84
*SD*	0.75	0.74	0.78	0.79	0.71	0.76	1.04	1.15	1.13	1.13	1.03	1.04	0.73	0.62	0.48	0.86	0.84	0.66	0.68	0.65	0.55	10.44	10.25	0.57	0.62	0.58	0.70

^*Note:* Statistically significant correlations (*p* <.05) are bold. W1–6 = Wave 1 through Wave 6 during G2 adolescence; YA = young adulthood; A = G2–G3 Wave A; B = G2–G3 Wave B.^

### Testing the G2–G3 parenting model

When fitting the G2-G3 parenting model, we initially found that the random intercepts for the family climate and adolescent social problem-solving skills were correlated (*r* = .68, *p* <.001) and, as a result, our model demonstrated suppressive effects from family climate to Wave A parental warmth. Specifically, our results indicated a significant and negative regression coefficient (suggesting that a positive family climate promoted lower levels of Wave A parental warmth), even though the bivariate correlation between these constructs was not significant. Thus, we constrained this path to zero. Similarly, the YA measures (relationship violence and couple problem-solving) were also correlated (*r* = −.46, *p* <.001), and our results indicated a significant and positive regression coefficient suggesting that YA relationship violence was positive associated with G2–G3 parental warmth, despite non-significant bivariate correlations. Thus, we constrained this path to zero. As a follow-up test, we recomputed the model, omitting the parental warmth variable from the model. This model yielded a consistent pattern of results to the constrained model, suggesting that these path constraints better reflect the data.

**Figure 2.**
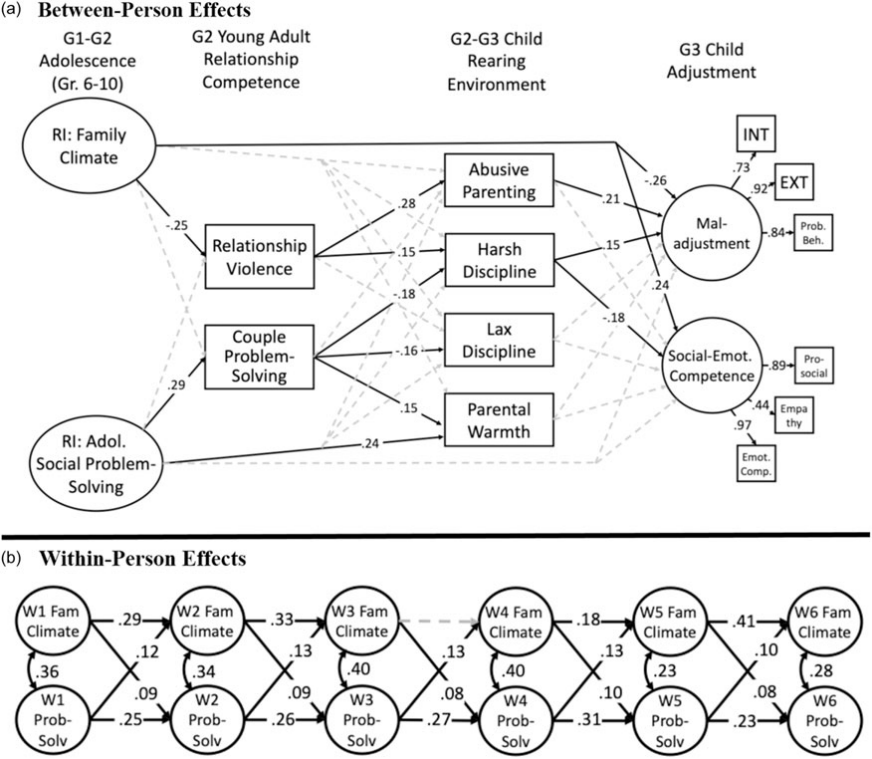
Developmental model evaluating effects on G2–G3 parenting and G3 child adjustment. (**a) Between-person effects**. (**b) Within-person effects**. *Note:* Fit: χ^2^(265) = 573.63, *p* <.01, CFI = 0.93, TLI =0.90, RMSEA = 0.0545 [90% C.I.: 0.048–0.060]. Solid lines were statistically significant, *p* <.05. RI = Random Intercept; INT = Internalizing Problems; EXT = Externalizing Problems; Emot. Comp. = Social and Emotional Competence. Covariates included in model, not pictured: sex of G2 parent, family income, G3 child age. For ease of presentation, some paths were not included in Figure 2a: covariances were estimated within each developmental period; paths regressing G3 child outcomes on G2 romantic competence also were estimated.

When we fit this modified G2-G3 parenting model to the data, we found that model fit was adequate, where *χ* ^2^(253) = 554.358, *p* <.01; CFI = 0.93; TLI = 0.90; RMSEA = .055 (90% C.I.:.049–0.061). We then evaluated the RI-CLPM using a step-wise procedure to determine whether autoregressive effects, within-occasion covariances, or cross-lagged paths could be constrained to be equal over time (Mulder & Hamaker, 2021). The fully constrained model resulted in significant deterioration of model fit (*χ* ^2^ (20) = 48.543, *p* = .0004). We then tested each block of paths (e.g., within-occasion covariances, autoregressive effects for family climate) separately to determine which caused model fit deterioration. This process identified constraints on within-occasion covariances and autoregressive effects for family climate as drivers of model fit change. When these two blocks were freely estimated, leaving the rest of the constraints in place the model no longer had different fit with the data than a freely estimated model, *χ* ^2^(12) = 19.278, *p* = .08. Therefore, the final model retained constrained paths in which adolescent social problem-solving autoregressive paths were equal over time, cross-lagged paths for family climate predicting social problem-solving were equal over time, and social problem-solving paths predicting family climate were equal over time. The final model and fit indices are presented in Figure 2; covariates included in the model but not depicted in the figure are presented in Table 3. In this figure, statistically significant paths (*p* < .05) are represented as solid lines, and dashed lines were not statistically significant. Non-significant paths from variables two periods before (e.g., YA romantic competence to G3 adjustment) are not presented but were estimated in the models. All contemporaneous variables were correlated but are not presented in the figure for clarity.

**Table 2.** Indirect effects (standardized beta coefficients)

	Indirect effect
Model #1: G2–G3 Parenting Model	
Indirect Effects from G1 Adolescence to G2–G3 Parenting	
G2 Social Prob. Solv. → YA Couple Prob. Solv. → G2–G3 Warmth	0.05*
G2 Social Prob. Solv. → YA Couple Prob. Solv.→ G2–G3 Harsh Disc.	−0.05*
G1–G2 Fam. Clim. → YA Rel. Viol. → G2–G3 Abusive Par.	−0.07*
Indirect Effects from G2 Young Adult Relationships to G3 Adjustment	
YA Rel. Vio. → G2–G3 Abusive Parenting → Maladjustment	0.06**
Indirect Effects from G1 Adolescence – G3 Adjustment	
G1–G2 Fam. Clim. → YA Rel. Viol. → G2–G3 Abusive Par.→ G3 Maladj.	−0.02*
Model #2: G2–G3 Family-Level Functioning Model	
Indirect Effects from G1 Adolescence to G2-G3 Family-Level Functioning	
G1–G2 Fam. Clim. → YA Rel. Viol. → G2–G3 Fam. Con.	−0.05*
Indirect Effects from G1 Adolescence – G3 Adjustment	
G2 Social Prob. Solv. → G2–G3 Family Routines → G3 SE Competence	0.05^ns^

^ns^*p* = .07. *p <0.05. **p <.01.

*Note:* Fam. Clim. = Family Climate; Social Prob. Solv. = Social Problem-Solving, Couple Prob. Solv. = Couple Problem-Solving Skills; Rel. Viol. = Relationship Violence, Lax Disc. = Lax Discipline; Harsh Disc. = Harsh Discipline; Abusive Par. = Abusive Parenting, Fam. Con. = Family Conflict; Maladj. = Maladjustment; SE Competence = Social–Emotional Competence.

**Table 3.** Covariate effects to wave A/B (standardized beta coefficients)

	Wave A				Wave B	
Model #1	Warmth	Lax	Harsh	Abusive	Maladj	SE Comp
Parent sex	−0.34***	−0.04	−0.01	0.00	0.05	0.02
Family income	0.05	−0.12*	0.03	−0.06	−0.13*	0.08
Child age	−0.09	−0.11*	0.15**	−0.07	−0.03	0.22***
Model #2	Conflict	Cohesion	Routines		Maladj	SE Comp
Parent sex	0.08	−0.11*	−0.16**		−0.01	0.06
Family income	−0.14*	0.11*	0.08		−0.12*	0.06
Child age	−0.03	0.01	−0.10*		−0.03	0.21***

*p <0.05. **p <0.01. ***p <0.001.

In the cross-lag portion of the model, we observed consistently statistically significant stability paths, suggesting moderate inertia across occasions. Additionally, covariances within measurement occasions were statistically significant, representing consistent corresponding change between family climate and social problem-solving skills. At occasions when the family climate was more positive than usual, adolescents’ social problem-solving also was higher than usual. Cross-lagged effects were evident consistently across adolescence, indicating bidirectional, within-person effects: times of better family climate than usual predicted increases in social problem-solving skills at the next occasion; likewise, times of higher social problem-solving than usual predicted increases in the quality of the family climate at the next measurement occasion.

Turning to the long-term cascading effects, predictions from the random intercepts indicated that a more positive G1–G2 family climate was associated with reduced risk for YA relationship violence, and greater G2 adolescent social problem-solving skills predicted more effective YA couple problem-solving skills. In turn, G2 YA relationship violence was associated with higher risk for G2–G3 harsh discipline and abusive parenting; G2 YA couple problem-solving was associated with lower risk for harsh discipline, lax discipline, and higher parental warmth. Interestingly, a direct effect from adolescent social problem-solving skills to higher levels of G2–G3 parental warmth was evident, accounting for effects from YA romantic competence. Predicting G3 child outcomes, harsh discipline and abusive parenting were uniquely associated with greater G3 maladjustment; harsh parenting also was associated with poorer G3 social–emotional competence. In addition, the G1-G2 family climate had a direct negative association with G3 maladjustment and a positive association with G3 social–emotional competence. We then estimated indirect effects, presented in Table 2. Adolescent social problem-solving skills had an indirect effect on G2–G3 parental warmth and harsh discipline, via YA couple problem-solving skills. The G1–G2 family climate had an indirect effect on G2–G3 abusive parenting; further the indirect effect extended from family climate to G3 child maladjustment.

### Testing the G2–G3 family-level functioning model

**Figures 3.**
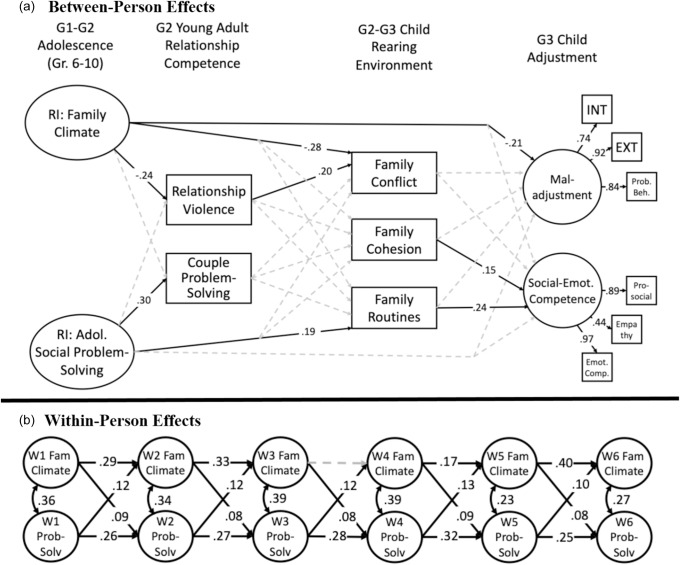
Developmental model evaluating effects on G2–G3 family-level functioning and G3 child adjustment. (**a) Between-person effects. (b) Within-person effects**. *Note:* Fit: χ^2^(249) = 508.28, *p* <.01, CFI = 0.93, TLI = 0.92, RMSEA = 0.051 [90% C.I.: 0.045–0.058]. Solid lines were statistically significant, *p* <.05. RI = Random Intercept; INT = Internalizing Problems; EXT = Externalizing Problems; Emot. Comp. = Social and Emotional Competence. Covariates included in model, not pictured: sex of G2 parent, family income, G3 child age. For ease of presentation, some paths were not included in Figure 3a: covariances were estimated within each developmental period; paths regressing G3 child outcomes on G2 romantic competence also were estimated.

The next model evaluated G2–G3 family-level functioning. As before, the overall model yielded adequate fit with the data: χ^2^(237) = 489.857, *p* <.01; CFI = 0.94; TLI = 0.91; RMSEA = 0.052 (90% C.I.:.045–0.058). We then evaluated whether constraints in the RI-CLPM portion of the model could arrive at a more parsimonious model. As in the parenting model, constraints on the autoregressive effects for social problem-solving, and cross-lagged effects in both directions were constrained to be equal over time, leaving family climate autoregressive paths and within-occasion covariances freely estimated resulted in a model with non-different fit from the freely estimated model *χ* ^2^(12) = 18.421, *p* = .10). The final model and fit indices are presented in Figure 3; covariates included in the model but not depicted in the figure are presented in Table 3.

Results from the cross-lag portion of the model generally mirrored those from first model. Further, the pattern of results from G1–G2 family climate and adolescent social problem-solving with YA romantic competence also were consistent with the first model. Turning to effects on G2–G3 family-level functioning, only G2 YA relationship violence was associated with family conflict; all other links with YA romantic competence and G2–G3 family-level functioning were not statistically significant. However, a direct effect was significant, indicating that a more positive G1–G2 family climate was associated with lower risk for G2–G3 family conflict; and greater G2 social problem-solving skills in adolescence were directly associated with more consistent G2–G3 family routines.

G2–G3 family-level cohesion and routines each were uniquely associated with G3 social–emotional competence. However, none of the G2–G3 family-level indices were associated with G3 maladjustment. As observed in the first model, a more positive G1–G2 family climate was associated with lower risk for G3 maladjustment. Two tests of indirect effects were conducted. First, a statistically significant indirect effect was observed in which a more positive G1–G2 family climate was associated with lower levels of G2–G3 family conflict, via reduced YA relationship violence. The second indirect effect tested whether G2 adolescent social problem-solving skills was associated with better G3 social–emotional competence, via improved G2–G3 family routines; however, this pathway did not pass the threshold for statistical significance (*p* = .07).

### Evaluating PROPSER effects on the developmental cascades

We then tested between-person paths one at a time for moderation by the intervention condition. In doing so, two significant effects emerged in which paths differed for the intervention and control groups. In both cases, the moderation effects were consistent across Model 1 and Model 2. First the direct pathway from G1–G2 family climate to G3 social–emotional competence was significant for the intervention group (B = 0.36, SE = 0.12, *p* <.05 for Model #1; *B* = 0.30, SE = .12, *p* <.05 for Model #2), but not statistically significant for the control group (B = 0.11, SE = 0.13, *ns* for Model #1; *B* = 0.03, SE = 0.13, *ns* for Model #2). All beta coefficients are unstandardized.

Second, the path from adolescent social problem-solving skills to YA couple problem-solving was significant for the intervention group (B = 0.42, SE = 0.11, *p* <.05 for Model #1 and #2) and non-significant for the control group (B = 0.18, SE = 0.11, *ns* for Model #1; *B* = 0.19, SE = 0.12, *ns* for Model #2). Probing this effect further, we examined indirect effects of G2 social problem-solving skills on G2–G3 parenting quality and G3 child adjustment. Tests of indirect effects revealed that G2 adolescent social problem-solving skills were indirectly linked with parental warmth (B = 0.07, *p* <.05) and with lax discipline (B = −0.12, *p* <.05), but not with harsh discipline (B = −0.08, *p* = .11). Extensions of these indirect effect tests to G3 child adjustment and social–emotional competence also were not statistically significant. All beta coefficients reported for these tests are unstandardized.

## Discussion

This study evaluated a long-term intergenerational cascade model in which family climate and adolescent social problem-solving skills in early adolescence (G1–G2) were linked with romantic competence (i.e., couple problem-solving and relationship violence) in young adulthood; in turn, romantic competence in young adulthood predicted the quality of the childrearing environment these adults provided to their offspring (G2–G3), and ultimately their offsprings’ (G3) maladjustment and social–emotional competence. In total, this study spanned approximately 20 years, highlighting pathways of continuity across these developmental periods.

Starting in the adolescent developmental period, our findings suggest that adolescent social problem-solving skills and family climate are interconnected processes. Across all six measurement occasions in early and middle adolescence, we observed corresponding change: at times when the family climate was more positive than usual levels across the six waves, adolescents also exhibited increased social problem-solving skills. Moreover, prospective cross-lagged paths indicate that these processes mutually shape each other over time. Across both models, our findings provide support for a dynamic interplay between family and adolescent functioning (Fosco et al., 2016; Mak et al., 2018). In addition, these findings suggest that interventions targeting adolescent skills or family relationship quality would have the most potent cross-over effects during the early-to middle-adolescent period, aligning with the timing of several existing family-and adolescent-focused prevention programs (Botvin & Griffin, 2004; Spoth et al., 2004).

### Adolescent developmental precursors to YA Romantic competence

Turning attention to the developmental cascades, our analyses also revealed long-term links between the adolescent period and later outcomes in adulthood. Both adolescent social problem-solving skills and family climate had unique, specific prospective relations with YA romantic competence. Across both analytic models, a consistent pattern emerged in which adolescents in families with more positive climates were at lower risk for YA relationship violence, but no link was evident for couple problem-solving; whereas adolescents who exhibited better social problem-solving skills reported more effective couple conflict problem-solving skills, but no relation was found with interpersonal violence. These findings support two of the hypothesized developmental pathways described in the DEARR model (Bryant & Conger, 2002). The first pathway – linking G1–G2 family relationships with YA relationship violence – adds to accumulating evidence highlighting the long-term implications of family relationship quality for reducing risk for YA relationship violence, even when accounting for important adolescent individual skills. In the current study, the enduring effect of family relationship quality was evident, over and above adolescent social problem-solving skills; but past work has also found that this association holds when accounting for adolescent assertiveness (Xia et al., 2018), and adolescent hostile-aggressive behavior (Fosco et al., 2016). Beyond the postulates of the DEARR model, our findings also fit with ideas expressed in a family disengagement risk model during adolescence (Fosco & LoBraico, 2019), which documents normative declines in parental involvement and parenting quality (Dishion et al., 2004; Lippold et al., 2016, 2018), and family-level relationship quality (Fosco et al., 2014) during this period. All of these studies converge on the ideas that the family is an important context for modeling and reinforcing adolescents’ interpersonal behavior (Ackerman et al., 2011; Patterson, 2015) and the importance of maintaining family relationship quality during this period to ensure optimal development in late adolescence and young adulthood.

The current findings also support a second pathway of the DEARR model, in which adolescent’s social problem-solving skills set the stage for more effective couple problem-solving practices in later romantic relationships. Our findings support a developmental perspective that the underpinnings of healthy young adult romantic relationships are evident in adolescents’ effective use of social problem-solving skills generalize to a healthier romantic relationship process in young adulthood. In our study, these effects were only observed for adolescents who were randomized to the PROSPER intervention condition. Many community-based or school-based interventions, including the ones utilized in PROSPER, target these skills (Botvin & Griffin, 2004; Das et al., 2016; Hawkins et al., 1991; Spoth et al., 2022). Our data suggest that PROSPER-delivered interventions may strengthen the link between adolescent social problem-solving skills and young adult couple problem-solving skills; in turn, these adolescent skills exert effects out to G2-G3 parental warmth and lax discipline practices. Further evaluation of these effects on adolescent social problem-solving skills are warranted, both to understand the broader implications for young adult developmentally salient outcomes (Roisman et al., 2004), and for potential cross-context effects on family relationship quality and in turn YA romantic competence (LoBraico et al., 2022).

### Cascading effects from G1–G2 in adolescence to G2–G3 outcomes

We also evaluated links between YA romantic competence, the G2–G3 childrearing environment, and ultimately with G3 child adjustment. These links were evaluated in two models with different aspects of the childrearing environment: the first focusing on parenting practices, and the second model focusing on family-level functioning. In the first model, YA romantic competence predicted all four parenting processes: relationship violence was associated with abusive parenting and harsh discipline, while couple problem-solving skills were associated with warmth and harsh and lax discipline. Importantly, for each of these links, we found statistically significant indirect effects of social problem-solving or the family climate in adolescence on parenting quality via YA romantic competence. In the second model, including G2–G3 family-level functioning, the only significant association was found between G2 YA relationship violence and G2–G3 family conflict, stemming from G1–G2 family climate.

The G2–G3 childrearing environment also had prospective relations with G3 child adjustment. In the parenting quality model, abusive parenting and harsh discipline each were associated with child psychological maladjustment, and harsh discipline additionally predicted social–emotional competence. Lax discipline and parental warmth were not associated with G3 adjustment outcomes. In the family-level functioning model, G2–G3 family cohesion and family routines were each associated with G3 children’s social–emotional competence; family conflict was not associated with either outcome. These findings underscore the multidimensional nature of parenting and family-level functioning, and the unique pathways by which families confer risk for child maladjustment or promote social–emotional competence. Our findings suggest that harsh, abusive parenting is a robust risk factor for children’s psychological maladjustment and lower levels of social–emotional competence. These findings are aligned with work on coercion theory (Patterson, 1982; Patterson, 2015): harsh and aversive parenting strategies for managing children’s behavior promote poorly regulated, less prosocial, and more maladjusted children.

In our second model, our findings underscore links between family-level routines and cohesion and children’s social–emotional competence. These findings build on existing research suggesting that family-level cohesion – reflecting close relationships, positive affect, and support – promotes children’s self-regulation and prosocial behavior (Chen et al., 2022; Cheng et al., 2024; Fosco & Grych, 2013). Similarly, households characterized by predictable routines also support children’s social–emotional development (Fiese et al., 2002; Ren & Fan, 2019). These findings point to the importance of positive, organized, predictable family interactions as nutriments of children’s social–emotional competence.

### Linking past and future generations: Indirect effect tests from G1 to G3

Our study evaluated two indirect pathways linking factors in G1–G2 adolescence to G3 child adjustment. The first pathway was statistically significant, despite being tested across three paths, linking four variables. This pathway linked interpersonal processes in which a positive G1–G2 family climate was associated with reduced risk for YA relationship violence, which in turn was associated with abusive parenting; G2–G3 abusive parenting was then associated with elevated risk for children’s maladjustment. These findings extend prior work documenting how the family climate in adolescence predicts’ YA’s self-regulation, problematic hostility, and romantic relationship violence (Fosco et al., 2012; Fosco et al., 2016). Our findings suggest a long-term sequenced transmission of family interaction patterns, established in adolescence, shaping YA romantic relationships, then G2–G3 parenting quality, and ultimately G3 child adjustment. In many ways, this cascading pathway reflects Elder’s (1994) concept of “linked lives” suggesting intergenerational interdependence in which the patterns of socialization and behavioral transmission of one generation is passed on to the next. We would also note that although less focused on three-generational transmission, structural family systems theory (Minuchin, 1974) also posits that interactional patterns, established in the family of origin are replicated in other intimate relationships. Together, these findings emphasize the power of family relationships in adolescence for the life course, and point to unique opportunity of family-centered preventive interventions that can have an impact on the family climate during this important developmental period (e.g., Redmond et al., 2009; Van Ryzin & Nowicka, 2013).

The second indirect pathway was tested in which adolescent social problem-solving, which was directly associated with G2–G3 family routines, and in turn, G3 social–emotional competence. It was interesting to note that there was a direct link between adolescent social problem-solving skills and next generation family routines, bypassing the young adult romantic relationships. This direct effect raises interesting questions about how social problem-solving skills that are honed during adolescence are generalized when raising the next generation, where parents identify challenging times of day when planful organization (e.g., morning or bedtime routines) help reduce parenting challenges. However, it is important to note that in our sample, the indirect effect from adolescent social problem-solving to G3 social–emotional competence did not achieve statistical significance. It is possible that our findings are limited by statistical power to detect the indirect effect, and future work may evaluate this linkage; however, in the current study, the indirect effect should not be interpreted substantively.

### Direct effects from adolescence to G3 adjustment

A surprising finding emerged in our analyses in which the G1–G2 family climate was directly associated with G3 maladjustment and social emotional competence, even when accounting for G2 young adult romantic competence and G2–G3 childrearing environment factors. Specifically, G3 children who had parents that benefitted from a more positive (G1–G2) family climate in adolescence exhibited lower risk for psychological maladjustment across both models. This direct effect suggests that there may be other important pathways of intergenerational transmission that were not captured in this study, calling for future studies to identify these uncharacterized pathways. We cannot rule out heritable genetic influences underlying this direct effect – or indeed any association in our developmental analyses. However, from an environmental influence perspective, a linked lives framework (Elder, 1994) informs speculation that a more positive G1–G2 family climate may help promote greater economic advantage for G2 life course trajectories by promoting greater family investment, supporting academic and occupational success, ultimately supporting the socio-economic status of the G2–G3 household. This pathway, driven by social–economic status is not accounted for in our analyses. A second possibility may be that our findings reflect an “enduring family influence,” derived from work on attachment theory (Fraley et al., 2013; Roisman & Fraley, 2013). In this model, family relationship quality may support a secure attachment, which is then carried forward into adulthood, unmediated by intervening family and life experiences. Perhaps our findings reflect an untested pathway in which a positive family climate in G1–G2 adolescence carries forward into attachment processes that then play out in the G2–G3 childrearing environment. The current study offers new answers and raises new questions about the intergenerational transmission from past to future generations.

Again, the PROSPER interventions appear to have tempered the direct effect of the relation between the G1–G2 family climate and G3 social–emotional competence. In our study, this direct, intergenerational link was observed for the intervention group but not the control group in both models. Although a more nuanced evaluation of these processes may be possible in future work, the current results indicate that evidence-based universal preventive interventions may strengthen the salience of positive, organized family interactions in adolescence, setting the foundation for interpersonal patterns that support better social–emotional competence in the G3 children.

### Limitations

As is common in intergenerational research, our sample reflects those participants in the original PROSPER community-randomized trial that had children at the time of our assessment. By some standards, these families may represent parents who had children at an age earlier than population norms; moreover, our sample includes parents of children of a wide age range. Thus, measures of parenting and child outcomes were intentionally general in nature, not allowing for a more precise, developmentally-specific assessment of these processes. Additionally, the PROSPER community-randomized trial was conducted in rural and semi-rural areas of Pennsylvania and Iowa, resulting in a primarily White sample, limiting generalizability to other families of racial and geographic diversity. Although our study represents a long-term longitudinal design, we were limited to mono-reporter methods, which may inflate the degree to which variables are correlated in our models. Last, our sample is likely underpowered to reject the null hypotheses (Mulder, 2023), and thus may have failed to detect significant effects. Future research, with larger intergenerational samples, would offer valuable replication of our findings.

## Conclusions

This study represents a 20-year longitudinal study capturing a developmental cascade that links G1–G2 family climate and adolescent social problem-solving skills as foundational processes for YA romantic competence; in turn, YA romantic competence predicted G2–G3 parenting and family-level relationship quality and ultimately G3 child adjustment. Our findings support two key, statistically significant pathways of intergenerational transmission. The first pathway indicates that a more positive family climate in G1–G2 adolescence was associated with reduced risk for YA relationship violence; in turn lower levels of relationship violence were associated with reduced risk for G2–G3 abusive parenting, ultimately reducing risk for G3 child maladjustment. The second finding suggests that there are direct links between the G1–G2 family climate and G3 child maladjustment risk, which may be explained by untested intervening processes. Finally, our results revealed that evidence-based universal prevention programs, delivered through PROSPER, may amplify the benefits of adolescent social problem-solving skills for young adult couple relationship functioning, and the long-term transmission of a positive G1–G2 family climate for G3 social–emotional competence.

## Data Availability

Data and code are available from the corresponding author.

## Pre-registration statement

This study was not pre-registered.
